# Sexual dysfunction during the late postpartum period: prevalence and associated factors

**DOI:** 10.3389/fpsyt.2025.1675863

**Published:** 2026-01-19

**Authors:** Juan Miguel Martínez-Galiano, Nuria Infante-Torres, Inmaculada Ortiz-Esquinas, Ana Rubio-Álvarez, Ana Ballesta-Castillejos, Antonio Hernandez-Martinez

**Affiliations:** 1 Department of Nursing, University of Jaén, Jaén, Spain; 2 Consortium for Biomedical Research in Epidemiology and Public Health (CIBERESP), Madrid, Spain; 3 Hospital Virgen de Altagracia, Manzanares, Spain; 4 Hospital Universitario Reina Sofia, Córdoba, Spain; 5 Hospital Universitario de Torrejon, Torrejón de Ardoz, Spain; 6 Castilla-La Mancha Health Research Institute (IDISCAM), Albacete, Spain; 7 Department of Nursing, Physiotherapy and Occupational Therapy, Faculty of Nursing, Albacete, University of Castilla-La Mancha, Ciudad Real, Spain; 8 Department of Nursing, Physiotherapy and Occupational Therapy, Faculty of Nursing, Ciudad Real, University of Castilla-La Mancha, Ciudad Real, Spain

**Keywords:** female sexual function, postpartum, puerperium, sexual dysfunction, sexuality, obstetric violence, pregnancy

## Abstract

**Background:**

Addressing women’s sexuality during the perinatal period receives little attention in research, and few studies are available. Furthermore, there is no consensus on the factors that influence the development of sexual dysfunction in the late postpartum period.

**Objective:**

Determine the prevalence of sexual dysfunction in women during the late postpartum period and the factors associated with it.

**Method:**

A observational study was conducted with women who had given birth in Spain in 2024. Information was collected on sociodemographic variables, personal history, and sexual function. Mean differences (MD), adjusted mean differences (aMD), odds ratios (OR), and adjusted odds ratios (aOR) with their respective 95% confidence intervals (CI) were calculated, as appropriate. All analyses were performed using the statistical program SPSS 29.0.

**Results:**

A total of 341 women participated. The prevalence of sexual dysfunction (FSFI scores <26.5) was 64.5%. The woman’s age (aOR: 1.10; 95%CI: 1.03-1.18), breastfeeding between 3 and 6 months postpartum (aOR: 3.34; 95%CI: 1.72-6.50), quality of life (SF-12) (aOR: 0.97; 95%CI: 0.95-0.99), and the WAST (aOR: 1.48; 95%CI: -1.13-1.93) are factors that influence the development of sexual dysfunction in the late postpartum period.

**Conclusion:**

The prevalence of sexual dysfunction in the medium term after childbirth is high. Younger age, non-breastfeeding status, good quality of life, and intimate partner relationships without violence are less likely to develop sexual dysfunction and have better sexual function.

## Introduction

### Statement of significance

**Table:** 

Issue	Women’s sexuality during the perinatal period has been under-researched, especially in the late postpartum stage, and there is no consensus on contributing factors to sexual dysfunction.
What is Already Known	Previous studies indicate that postpartum sexual dysfunction is common, but few clearly identify the associated factors in the medium term after childbirth
What this Paper Adds	Evidence of a high prevalence of sexual dysfunction in the late postpartum period and identification of influencing factors such as maternal age, breastfeeding, quality of life, and intimate partner violence

Sexual health encompasses more than just the absence of disease (1), as conceptualized by the World Health Organization (WHO), which also considers sexuality as a central aspect of a woman’s quality of life and a fundamental parameter of her physical, psychological, and social well-being (2).

Addressing sexuality is one of the aspects that receives the least attention from health services and research, even at such a crucial stage as the perinatal period, when sexuality is significantly affected (3, 4). Nor is the attention and care that women’s sexual health should receive considered (5).

Sexual dysfunction makes sexual relations difficult or even impossible and can occur at any stage of the sexual act (6). The number of women with these problems during the perinatal stage is high (7, 8), although the figures vary depending on the study population or the different trimesters of pregnancy or the postpartum period (9, 10).

Various factors have been identified as associated with sexual dysfunctions in women during the perinatal stage. These factors may be sociodemographic, such as age (7) or being a potential victim of gender-based violence (11), personal history, such as the presence of pathology (12), obstetric factors, such as parity (13) or type of birth (14), or factors related to the care received, such as the woman’s positive birth experience (15) or the type of suture used for the perineal injury sustained during childbirth (16).

The presence of sexual dysfunction not only entails all the discomforts inherent to these disorders but has also been associated with a greater presence of other problems, such as mental health-related pathologies (17).

The studies that have been identified are scarce, and there have been recent social and cultural changes that could influence the results. Furthermore, no consensus has been reached regarding many of the factors. Identifying these factors can guide the development of prevention strategies, management strategies, and early detection of the problem. Therefore, the aim was to determine the prevalence of sexual dysfunction in women in the medium term, during the late postpartum period, and the factors associated with it.

## Methods

A prospective observational study was carried out in 2024 with postpartum women who gave birth in Spain.

### Population and study subjects

The study population consisted women who had given birth in Spain. The following exclusion criteria were used to select the study subjects: women under 18 years of age, those who did not speak or understand the Spanish language (language barrier), and those diagnosed with a mental illness before pregnancy. The inclusion criteria included a period of 6–9 months since birth.

The maximum modeling criterion was used to estimate the sample size, which requires the inclusion of 10 subjects for each independent variable (18). Considering that the prevalence of risk for sexual dysfunction may reach up to 68% (19), a minimum of 200 women at risk for sexual dysfunction was required to include 20 independent variables in the model.

The women were recruited consecutively.

### Information sources

A self-developed, previously piloted questionnaire was used for data collection (**Supplementary Material**). This questionnaire was distributed to associations related to pregnancy, birth, and postpartum care, as well as to breastfeeding support groups throughout Spain. Sociodemographic variables, obstetric history, variables of the most recent birth, obstetric practices carried out, and neonatal outcomes were included.

Various tools were included in this questionnaire:

- Childbirth Abuse and Respect Evaluation- Maternal Questionnaire” (CARE-MQ) (20), version 2. This tool consists of Likert-type questions about different practices and/or situations that can be related to abuse and disrespect during childbirth. Possible responses are: “It did not occur during my birth” (0 points), “It occurred, but it did not affect me” (1 point), “It occurred and it affected me a little” (2 points), and “It occurred and it affected me a lot” (3 points). The total score ranged from 0 to 60 points. Scores can be categorized according to the percentile distribution (≤50th percentile, 51st-75th percentile, 76th-90th percentile, >90th percentile). The tool showed adequate internal consistency and excellent test-retest stability.
- Perinatal Post-Traumatic Stress Disorder Scale (PTSD) Questionnaire (PPQ). The risk of PTSD was assessed using the Perinatal Post-Traumatic Stress Disorder Questionnaire (PPQ). This questionnaire has been validated and used in a population similar to that of the study. This tool consists of 14 questions with Likert-type responses with scores ranging from 0 to 56 points (21). We considered a high-risk score for post-traumatic stress disorder as a score equal to or greater than the 90th percentile of its distribution.
- Quality of Life Questionnaire: The women’s quality of life was assessed using the SF-12 questionnaire (22). This questionnaire consists of a set of 12 items on HRQoL. The SF-12 version presents eight domains: physical functioning, physical role, bodily pain, general health, vitality, social functioning, emotional role, and mental health. The total quality of life score is obtained by summing the two subscales, physical health and mental health (23).
- Edinburgh Postnatal Depression Scale (EPDS). This scale consists of 10 self-reported items and is used to detect postpartum depression specifically. It has been validated in the Spanish postpartum population (24).

### Data collection

Data were collected in the first 2 months after birth and analyzed to address other objectives. At 6–9 months, we contacted the participants from the first phase again. At this time point, we assessed their risk of intimate partner violence and their sexual function using the following validated tools:

- WAST Short Form. The WAST Short Form consists of two items with three response options. It has good psychometric properties in both its original version and its validated Spanish version, with a sensitivity of 91.4% and a specificity of 76.2% (25).
- The Female Sexual Function Index (FSFI) (26) is designed to assess different facets of female sexual function. It consists of 19 questions grouped into six domains (lubrication, orgasm, desire, satisfaction, pain, and arousal). Responses are scored on a scale from 0 (not at all or almost not at all) to 5 (always or almost always). The score for each domain is multiplied by a factor, and the final result is the arithmetic sum of the domains. The highest total score is 45, ranging from 5-45. A decrease in these scores indicates a decline in sexual function. The cutoff point below which sexual dysfunction is considered is a score equal to or less than 26.5 points (27). This tool has also been validated in Spain (28).

### Statistical analysis

First, descriptive statistics were performed using absolute and relative frequencies for categorical variables and means with standard deviations for quantitative variables.

Next, the relationship between the time to return to sexual intercourse and sexual function (FSFI) was analyzed using the linear trend analysis of variance (ANOVA) test.

In the next step, the relationship between various sociodemographic and clinical factors measured at the first cutoff and the impact on sexual dysfunction (FSFI scores) at six months of follow-up was determined. The only independent variable included from the second cutoff was the Woman Abuse Screening Tool (WAST) scores. WAST is a validated instrument used in healthcare settings to identify domestic abuse and intimate partner violence in women. In this case, mean differences (MD) and adjusted mean differences (aMD) with their respective 95% confidence intervals (CI) were determined using multiple linear regression.

Finally, the relationship between various sociodemographic and clinical factors measured at the first cutoff and the impact on the risk of sexual dysfunction (FSFI scores <26.5) at six months of follow-up was determined. In this case, odds ratios (OR) and adjusted odds ratios (aOR) with their respective 95% confidence intervals (CI) were determined using binary logistic regression. Missing data were handled using listwise deletion. All analyses were performed using the statistical program SPSS 29.0.

### Ethical aspects

Approval was obtained from the clinical research ethics committees of the institutions where it was conducted. All participants received written information about the study and signed informed consent before participating.

## Results

### Sample characteristics

A total of 341 women participated, with a mean age of 33.8 years (SD = 4.23 years), 80.1% (273) of whom were primiparous. In 56.3% (192) of the cases, labor was induced, and 57.2% (195) resulted in a normal vaginal birth. Regional analgesia was used in 73.9% (251), and 15.5% (53) of the newborns were admitted to a neonatal unit. The remaining characteristics of the sample are shown in **Table 1**.

**Table 1:** Characteristics of the sample.

Variable	N (%) N=341	Variable	N (%) N=341
**Maternal age** Mean (SD)	33.8 (4.23)	**Regional analgesia**	
**Income**		No	90 (26.4)
< 1000 euros	6 (1.8)	Yes	251 (73.6)
1000–1999 euros	51 (14.9)	**General anesthesia**	
2000–2999 euros	110 (32.3)	No	325 (95.3)
3000–3999 euros	106 (31.1)	Yes	16 (4.7)
>= 4000 euros	68 (19.9)	**Type of birth**	
**Planned pregnancy**		Normal birth	195 (57.2)
No	29 (8.5)	Instrumental	65 (19.1)
Yes	312 (91.5)	Elective CS	11 (3.2)
**Antenatal education program**		Emergency CS	70 (20.5)
No	49 (14.4)	**Perineal trauma**	
Yes	292 (85.6)	No	263 (77.1)
**Fertility treatment (any medical assistance to achieve pregnancy)**		Yes	78 (22.9)
No	290 (85.0)	**Skin to skin**	
Yes	51 (15.0)	< 120 minutes	127 (37.2)
**Twin pregnancy**		Yes, and at least 120 minutes	214 (62.8)
No	339 (99.4)	**Neonatal Admission**	
Yes	2 (0.6)	No	288 (84.5)
**Gestational age**		Yes	53 (15.5)
Term	320 (93.8)	**Exclusive breastfeeding at discharge**	
Preterm	21 (6.2)	No	90 (26.4)
**Parity**		Yes	251 (73.6)
Primiparous	273 (80.1)	**Hospital readmission**	
Multiparous	68 (19.9)	No	331 (97.1)
**Induction of labor**		Yes	10 (2.9)
No	192 (56.3)	**CARE-MQ** Mean (SD)	8.3 (11.6)
Yes	149 (43.7)	**PPQ** Mean (SD)	12.4 (12.9)
**Problems during birth**		**EPDS** Mean (SD)	9.6 (6.2)
No	251 (73.6)	**SF-12** Mean (SD)	69.6 (19.3)
Yes	90 (26.4)	**WAS** Mean (SD)	1.0 (1.01)
		**BMI** Mean (SD)	24.2 (4.8)

### Prevalence of sexual dysfunction and its relationship with the time to return to sexual activity

**Figure 1:**
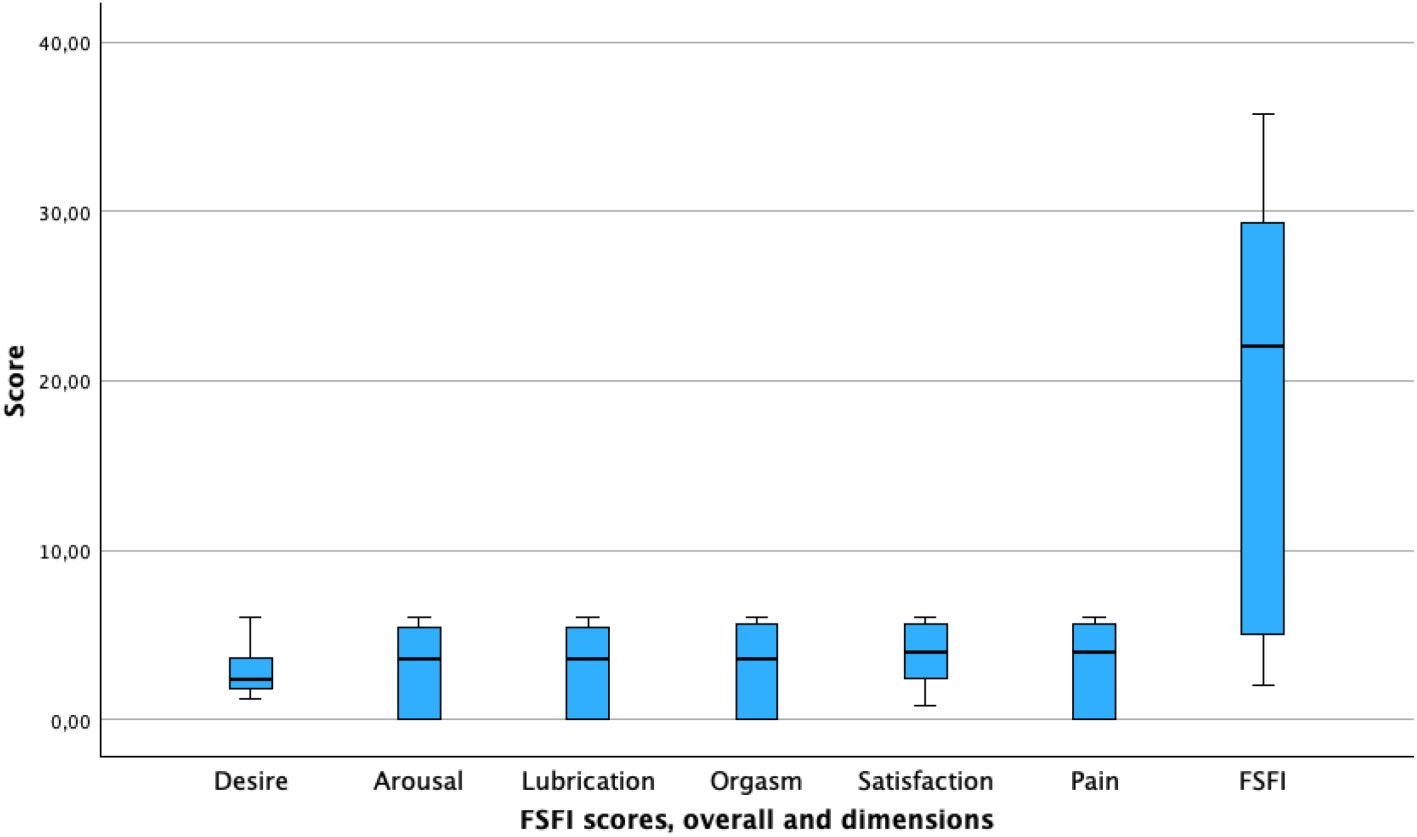
FSFI scores, overall and dimensions.

The prevalence of sexual dysfunction (FSFI scores <26.5) was 64.5% (220), with a mean score of 18.74 points (SD = 11.65). The most affected dimension was desire, with a mean score of 2.64 points (SD = 1.20), and the least affected dimension was satisfaction, with a mean score of 3.88 (SD = 1.68) (**Table 2**, **Figure 1**).

**Table 2:** Relationship between FSFI scores and the time of reestablishment of coital sexual relations.

FSFI dimensions		Time of reinitiating coital sexual relations following birth							
		Total (n=341)	< 1 month (n=14)	1–2 months (n=100)	2–3 months (n=76)	3–6 months (n=59)	> 6 months (n=18)	Not restarted (n=74)	P Value
Desire	Mean (SD)	2.64 (1.20)	3.99 (1.12)	2.92 (1.20)	2.86 (1.23)	2.67 (1.12)	2.70 (2.01)	1.75 (0.73)	**<0.001**
Excitation	Mean (SD)	3.00 (2.36)	4.52 (2.05)	3.72 (1.96)	3.68 (2.16)	3.53 (2.37)	3.15 (2.01)	0.56 (1.40)	**<0.001**
Lubrication	Mean (SD)	3.00 (2.43)	4.22 (2.01)	3.85 (2.09)	3.71 (2.09)	3.46 (2.37)	3.20 (2.30)	0.46 (1.45)	**<0.001**
Orgasm	Mean (SD)	3.07 (2.50)	4.31 (2.20)	3.74 (2.14)	3.73 (2.31)	3.88 (2.40)	3.24 (2.30)	0.56 (1.54)	**<0.001**
Satisfaction	Mean (SD)	3.88 (1.68)	4.86 (1.80)	4.22 (1.55)	4.37 (1.42)	4.26 (1.65)	4.42 (1.44)	2.31 (1.15)	**<0.001**
Pain	Mean (SD)	3.16 (2.52)	4.89 (2.14)	4.19 (2.05)	4.03 (2.23)	3.58 (2.15)	3.58 (2.15)	0.11 (0.74)	**<0.001**
SFSI Total	Mean (SD)	18.74 (11.65)	26.79 (10.54)	22.64 (9.85)	22.39 (10.27)	21.36 (10.91)	20.29 (9.62)	5.76 (5.67)	**<0.001**

Values in bold are statistically significant.

**Figure 2:**
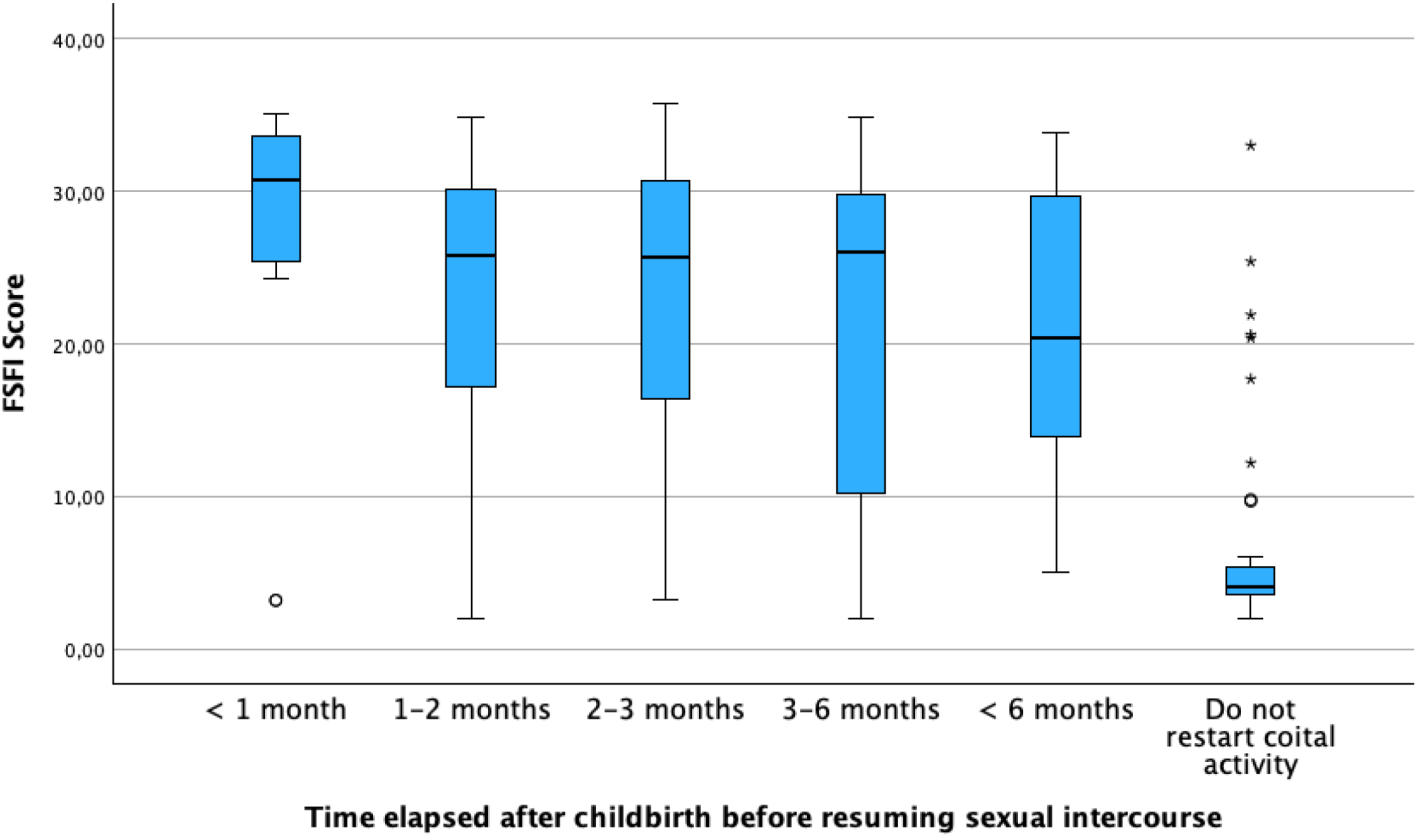
Relationship between FSFI scores and the time of reestablishment of coital sexual relations.

The relationship between the time to return to sexual activity and FSFI scores was then analyzed, revealing a statistically significant and linear association. Thus, the longer the time to return, the worse the scores for Sexual Function were, both overall and across all dimensions. (**Table 2**, **Figure 2**).

### Factors associated with FSFI scores

The following analysis identified factors associated with FSFI scores. Multivariate analysis showed a statistically significant relationship with the woman’s age (aMD: -0.47; 95%CI: -0.16 to -0.79), the use of fertility treatment (aMD: -3.92; 95%CI: -0.10 to -7.41), breastfeeding between 3 and 6 months postpartum (aMD: -4.53; 95%CI: -1.34 to -7.73), quality of life (SF-12) (aMD: 0.13; 95%CI: 0.04 to 0.22), and the risk of intimate partner violence (WAST) (aMD: -1.55; 95%CI: -0.31 to -2.73). Thus, younger women, who had not used fertility treatment, did not breastfeed, had better quality of life and without risk of intimate partner violence on average had better scores on the Sexual Function Index (**Table 3**).

**Table 3:** Bivariate and multivariate analysis Factors associated with sexual dysfunction FSFI score .

Variable	Bivariate analysis FSFI Total		Multivariate analysis FSFI Total	
	MD (95% CI)	MD (95% CI)	aMD (95% CI)	P Value
Age (per year)	**-0.60 (-0.31 to -0.88)**	**<0.001**	**-0.47 (-0.16 to -0.79)**	**0.003**
BMI (per point)	0.18 (-0.07 to 0.44)	0.161	0.15 (-0.10 to 0.39)	0.247
Fertility treatment (RC No)	**-6.00 (-2.58 to -9.43)**	**<0.001**	**-3.92 (-0.10 to -7.41)**	**0.028**
Time after birth (per month)	**0.38 (0.11 to 0.65)**	**0.007**	0.24 (-0.03 to 0.51)	0.082
Cesarean birth (RC vaginal)	-0.21 (-2.72 to 2.30)	0.870	1.51 (-1.35 to 4.37)	0.290
Parity (RC primiparous)	-0.78 (-3.88 to 2.33)	0.624	1.53 (-1.65 to 4.71)	0.325
Perineum intact (RC no)	1.21 (-1.33 to 3.74)	0.351	0.87 (-1.74 to 3.49)	0.507
Currently breastfeeding (RC no)	**-5.77 (-2.61 to -8.93)**	**<0.001**	**-4.53 (-1.34 to -7.73)**	**0.006**
EPDS (per point)	-0.09 (-0.29 to 0.10)	0.353	0.10 (-0.21 to 0.40)	0.530
CARE- MQ (per point)	-0.01 (-0.10 to 0.12)	0.893	-0.00 (-0.14 to 0.13)	0.976
PPQ (per point)	-0.03 ( -0.13 to 0.06)	0.522	0.02 (-0.14 to 0.18)	0.832
SF12 (per point)	**0.11 (0.05 to 0.18)**	**<0.001**	**0.13 (0.04 to 0.22)**	**0.005**
WAST (per point)	**-1.66 (-0.44 to -2.87)**	**0.008**	**-1.55 (-0.31 to -2.73)**	**0.014**

Values in bold are statistically significant.

### Factors associated with risk of sexual dysfunction

Finally, factors associated with the risk of sexual dysfunction (FSFI scores <26.5) were identified. Multivariate analysis showed a statistically significant relationship with the woman’s age (aOR: 1.10; 95% CI: 1.03-1.18), breastfeeding between 3 and 6 months postpartum (aOR: 3.34; 95%CI: 1.72-6.50), quality of life (SF-12) (aOR: 0.97; 95%CI: 0.95-0.99), and the WAST (aOR: 1.48; 95%CI: 1.13-1.93). Thus, younger women, those who did not breastfeed, and those with better quality of life and without risk of intimate partner violence had a lower risk of sexual dysfunction (**Table 4**).

**Table 4:** Bivariate and multivariate analysis. Factors associated with risk of sexual dysfunction.

Variables.	Risk of sexual dysfunction		Bivariate analysis		Multivariate analysis	
	No n (%) (N = 121)	Yes n (%) (N = 220)	OR 95% CI	p-value	aOR 95% CI	p-value
Maternal age Mean (SD)	32.7 (3.84)	34.4 (4.31)	**1.11 (1.05-1.17)**	**<0.001**	**1.10 (1.03-1.18)**	**0.005**
BMI Mean (SD)	24.6 (4.45)	24.0 (4.99)	0.98 (0.93-1.02)	0.304	0.37 (0.93-1.03)	0.369
Fertility treatment				**0.012**		0.146
No	111 (38.3)	179 (61.7)	1		1	
Yes	10 (19.6)	41 (80.4)	**2.54 (1.22-5.28)**		1.82 (0.81-4.09)	
Time after birth (months) Mean (SD)	8.1 (5.67)	7.6 (3.79)	0.98 (0.93-1.03)	0.332	1.00 (0.95-1.06)	0.908
Cesarean birth				0.521		0.934
No	72 (36.9)	123 (63.1)	1		1	
Yes	49 (33.6)	97 (66.4)	1.16 (0.74-1.82)		0.98 (0.53-1.79)	
Parity						
Primiparous	98 (35.9)	175 (64.1)	1	0.749	1	0.335
Multiparous	23 (33.8)	45 (66.2)	1.10 (0.63-1.92)		0.72 (0.37-1.41)	
Perineal trauma				0.239		0.210
No	93 (35.4)	170 (64.6)	1		1	
Yes	28 (35.9)	50 (64.1)	0.76 (0.49-1.20)		0.71 (0.41-1.22)	
Current breastfeeding				**<0.001**		**<0.001**
No	35 (56.5)	27 (43.5)	1		1	
Yes	86 (30.8)	193 (69.2)	**2.91 (1.66-5.11)**		**3.34 (1.72-6.50)**	
EPDS (per point) Mean (SD)	8.9 (6.60)	10.0 (6.00)	1.03 (0.99-1.07)	0.117	1.00 (0.93-1.06)	0.901
CARE- MQ (per point) Mean (SD)	8.3 (12.63)	8.3 (10.96)	1.00 (0.98-1.02)	0.984	0.99 (0.97-1.02)	0.672
PPQ (per point) Mean (SD)	11.5 (14.36)	12.9 (11.99)	1.01 (0.99-1.03)	0.358	0.99 (0.96-1.03)	0.901
SF12 (per point) Mean (SD)	74.7 (18.78)	66.7 (18.80)	**0.98 (0.96-0.99)**	**<0.001**	**0.97 (0.95-0.99)**	**0.003**
WAST (per point) Mean (SD)	0.8 (0.93)	1.17 (1.03)	**1.47 (1.16-1.86)**	**0.002**	**1.48 (1.13-1.93)**	**0.004**

Values in bold are statistically significant.

## Discussion

The number of women in the late postpartum period with sexual dysfunction was high; approximately 2 out of 3 of these women had some type of dysfunction. Desire was the most affected component, while satisfaction was the least. The longer the time elapsed from delivery to the resumption of sexual relations, the worse the sexual function. FSFI scores were higher in younger women, those who had not used fertility treatment to conceive, those who did not breastfeed, and those with good quality of life and without risk of intimate partner violence. This was reflected in the fact that women who were less likely to develop sexual dysfunction were younger, did not breastfeed, and those with good quality of life and without risk of intimate partner violence, with many of the factors coinciding.

Since this is a questionnaire, anamnestic and recall bias cannot be completely ruled out. Recruitment through breastfeeding associations and groups may introduce selection bias and limit generalizability; however, we do not believe that its influence, if any, on the results was significant. Likewise, potential confounding bias, inherent to the type of study conducted, cannot be ruled out, as can its potential influence on the results. However, the selection of study subjects and the multivariate analysis largely controlled for this potential confounding. All instruments used were validated in a population similar to the study (20, 21, 23–25, 28).

The prevalence of sexual dysfunction in women in the late postpartum period was high; however, this figure is 30 percentage points lower than what Oliveira et al. found in their study conducted in Brazil with women in the late postpartum period (10) and also lower than what other authors have reported (29). A lower percentage than that detected in our study was reported by Wassenaar et al. (15). Nonetheless, our results are in line with the percentage of women who had sexual dysfunction in the postpartum period reported in research conducted with Australian women (13) and other studies (19).

In agreement with Khajehei et al. (13), the most affected aspect of sexuality in our results was desire, also coinciding with other authors (30, 31). Contrary to what has been identified, several authors do not identify satisfaction as the least affected aspect (10, 30), but rather they identify orgasm and lubrication instead of satisfaction, although other authors did identify this as one of the subdimensions of sexual function with the highest scores in postpartum women (32).

The time that passes from birth until sexual relations are resumed emerged as a factor that is associated with a woman’s sexual function; the earlier they begin, the better the sexual function. This may be because when the women were asked, sometime could have passed since they restarted sexual activity, and these were better and had normalized, considering that the first sexual relations after childbirth are not at all or only somewhat pleasurable for women (33). Our results coincide with those of Khajehei et al., who also identified an association with the late resumption of postpartum sexual activity (13).

The age of women was one of the factors identified, finding that the older the woman, the greater the probability of sexual dysfunction and worse sexual function appearing. There is no consensus in the literature regarding this factor. There are authors who, in line with our results, have identified this (29). This may be due to the decrease in estrogen that occurs in women with increasing age. However, a majority of studies do not find age to be a factor associated with sexual dysfunctions and sexual function (15, 30, 34, 35).

Breastfeeding was associated with a higher likelihood of developing sexual dysfunction and worse sexual function in women during the late postpartum period. Various authors have identified this; however, some distinguish between exclusive breastfeeding and mixed breastfeeding (36–38).

A significant number of studies (11, 15, 39) report a high-quality relationship with a partner and a lack of intimate partner violence as factors associated with a lower prevalence of sexual dysfunction and better sexual function in women in the postpartum period, as also identified in our results.

Women with a good quality of life are less likely to develop sexual dysfunction and have better sexual function, as also reported by other authors (40).

Women who required medical care to achieve pregnancy were more likely to experience sexual dysfunction and worse sexual function in the late postpartum period. This is in line with what Park described, with women undergoing fertility treatments having fewer or reduced orgasms (41). Contrary to previous literature, depression, post-traumatic stress disorder, birth experience, and perineal injury showed no significant association with sexual dysfunction in this study (15, 42, 43).

Women’s sexual health in the perinatal stage should be addressed. In this regard, the implementation of strategies to identify women most likely to develop sexual dysfunction, addressing modifiable factors, and developing health education programs (or including this information in existing ones) can contribute to reducing the magnitude of this problem and its associated consequences.

## Conclusions

The prevalence of sexual dysfunction in the late postpartum period is high. Women who resume sexual activity early have better sexual function. Younger women, those who do not breastfeed their babies, and those with a good quality of life, and a relationship free of intimate partner violence, are less likely to develop sexual dysfunction and have better sexual function.

## Data Availability

The raw data supporting the conclusions of this article will be made available by the authors, without undue reservation.
