# Supplementary material for: Sexual dysfunction during the late postpartum period: prevalence and associated factors
Source: Front Psychiatry. 2026 Jan 19;16:1675863. doi: 10.3389/fpsyt.2025.1675863 (PMC12861907; doi:10.3389/fpsyt.2025.1675863)
Supplement: Supplementary file 1 [file Supplementaryfile1.docx]

### ****SUPPLEMENTARY MATERIAL: QUESTIONNAIRE ON PERINATAL EXPERIENCE, PERCEIVED OBSTETRIC VIOLENCE, AND POSTPARTUM MENTAL HEALTH****

**Adapted from the original questionnaire of the University of Castilla-La Mancha (2024).**

## ****SECTION 1.****

Below, please provide information about relevant social and demographic aspects.

**What is your age?***
(Write your age in years)
▢ _____

**In which of the following groups would you place your total monthly family income?***
▢ Less than €1,000
▢ Between €1,000 and €2,000
▢ Between €2,000 and €3,000
▢ Between €3,000 and €4,000
▢ More than €4,000

**Did you attend prenatal (maternal) education classes during your last pregnancy?***
Select one oval only.
▢ No
▢ Yes

**Was your pregnancy planned?***
▢ No
▢ Yes

**Was your baby born alive?***
▢ Yes
▢ No

Below we ask for information about your last pregnancy and childbirth.

**Please indicate how many times you have experienced the following:***

| **Process** | **None** | **One** | **Two** | **Three** | **More than three** |
| --- | --- | --- | --- | --- | --- |
| Number of pregnancies | ☐ | ☐ | ☐ | ☐ | ☐ |
| Number of miscarriages | ☐ | ☐ | ☐ | ☐ | ☐ |
| Number of cesarean sections | ☐ | ☐ | ☐ | ☐ | ☐ |
| Number of vaginal births | ☐ | ☐ | ☐ | ☐ | ☐ |

**How many weeks pregnant were you at the time of delivery?***
▢ Full-term (37 weeks or more)
▢ Preterm (less than 37 weeks)

**In this pregnancy, how many babies were you carrying?***
▢ One baby
▢ Two or more babies

**During your last pregnancy, did you experience any of the following problems?***

| **Problem** | **No** | **Yes** | **I don’t know** |
| --- | --- | --- | --- |
| High blood pressure (hypertension) | ☐ | ☐ | ☐ |
| Diabetes | ☐ | ☐ | ☐ |
| Threat of preterm labor (labor starting before 37 weeks) | ☐ | ☐ | ☐ |
| Other | ☐ | ☐ | ☐ |

If you marked “Other,” please specify: __________

**Did you need to use any assisted reproduction technique to become pregnant?***
▢ No
▢ Yes

**Was your labor induced?***
▢ No
▢ Yes

If you answered “Yes,” please indicate the reason if known: __________

**Did you use any type of analgesic method for pain relief?***

| **Method** | **No** | **Yes** | **I don’t know** |
| --- | --- | --- | --- |
| Epidural or spinal anesthesia | ☐ | ☐ | ☐ |
| General anesthesia | ☐ | ☐ | ☐ |

**Did you have an episiotomy (a cut made by the midwife or doctor in the perineum)?***
▢ No
▢ Yes

**Did you have any perineal tear (different from episiotomy)?***
▢ No
▢ Yes

### ****How did your childbirth end?*****

Select one oval.
▢ It was a normal (eutocic) birth
▢ It was an assisted birth using spatulas, vacuum extractor or forceps
▢ It was a planned cesarean section
▢ It was an emergency cesarean section

**Were you able to have skin-to-skin contact with your baby after birth for at least 120 minutes?***
▢ No
▢ Yes

### ****Was your baby receiving exclusive breastfeeding at hospital discharge?****

(If you had a home birth, indicate the type of feeding on the baby’s 3rd day of life.)
Select one oval.
▢ No
▢ Yes

**Did your baby have to be hospitalized?***
▢ No
▢ Yes

### ****After being discharged, did you have to be admitted to the hospital again?****

(If you had a home birth and later required hospital admission, please answer “Yes.”)
Select one oval.
▢ Yes
▢ No

### ****How many months ago was your last child born?****

(Write the number of months. For example, if your child was born one and a half months ago, write 1.5)

### ****Are you currently breastfeeding?*****

Select one oval.
▢ No
▢ Yes

## ****SECTION 2.****

**Childbirth Abuse and Respect Evaluation - Maternal Questionnaire (CARE-MQ)**

**Instructions:**
Below, please indicate whether any of the following situations occurred during your childbirth and, if so, how it affected you.
Please mark the option that corresponds in each case.

1. The professionals who attended my childbirth introduced themselves by name and profession.
    ▢ They did introduce themselves
    ▢ They did not introduce themselves, but it did not affect me at all
    ▢ They did not introduce themselves, and it affected me a little
    ▢ They did not introduce themselves, and it affected me a lot
2. The professionals explained the techniques and/or procedures to be performed (for example, inserting an IV, rupturing the membranes, administering medication, etc.), the reason for them, the alternatives, and the risks and benefits in an understandable way; and/or I was able to ask questions and choose among the proposed alternatives.
    ▢ Yes, they did
    ▢ No, but it did not affect me at all
    ▢ No, and it affected me a little
    ▢ No, and it affected me a lot
3. The professionals clearly explained the progress of my labor, my own health status, and that of my baby in an understandable way, and/or I was able to ask questions about any doubts that arose.
    ▢ Yes, they did
    ▢ No, but it did not affect me at all
    ▢ No, and it affected me a little
    ▢ No, and it affected me a lot
4. The professionals who attended me protected my privacy and intimacy (for example, by using screens or covering my intimate parts).
    ▢ Yes, they did
    ▢ No, but it did not affect me at all
    ▢ No, and it affected me a little
    ▢ No, and it affected me a lot
5. During vaginal examinations and/or procedures, there were more people present than necessary (other doctors, nurses, cleaning staff, etc.) or students (of nursing or medicine) were present without anyone asking my permission.
    ▢ Did not occur
    ▢ Occurred, but it did not affect me at all
    ▢ Occurred, and it affected me a little
    ▢ Occurred, and it affected me a lot
6. I was allowed to be accompanied by the person I chose throughout the entire childbirth process.
    ▢ Yes, I was allowed
    ▢ I was not allowed, but it did not affect me at all
    ▢ I was not allowed, and it affected me a little
    ▢ I was not allowed, and it affected me a lot
7. When I asked for help (to move, wash myself, relieve pain, etc.), I was attended to.
    ▢ Yes, I was attended to
    ▢ No, but it did not affect me at all
    ▢ No, and it affected me a little
    ▢ No, and it affected me a lot
8. I was helped with the care of my baby, with breastfeeding (either breast or bottle), and/or my questions were answered.
    ▢ Yes, I was helped and my questions were answered
    ▢ No, but it did not affect me at all
    ▢ No, and it affected me a little
    ▢ No, and it affected me a lot
9. The professionals respected my birth plan when possible, and when it was not possible, they explained the reason and we discussed an alternative.
    ▢ Yes, it was respected
    ▢ No, but it did not affect me at all
    ▢ No, and it affected me a little
    ▢ No, and it affected me a lot
10. I was scolded during childbirth, or when I asked questions, I was answered disrespectfully (with criticism, shouting, or insults).
     ▢ Did not occur
     ▢ Occurred, but it did not affect me at all
     ▢ Occurred, and it affected me a little
     ▢ Occurred, and it affected me a lot
11. I was frightened or intimidated verbally about the danger to me or my baby to make me accept certain practices I disagreed with, and they did not explain the reasons for them.
     ▢ Did not occur
     ▢ Occurred, but it did not affect me at all
     ▢ Occurred, and it affected me a little
     ▢ Occurred, and it affected me a lot
12. They spoke to me as if I were a child or ridiculed me.
     ▢ Did not occur
     ▢ Occurred, but it did not affect me at all
     ▢ Occurred, and it affected me a little
     ▢ Occurred, and it affected me a lot
13. I was criticized during childbirth for expressing my emotions (crying, shouting from pain, etc.).
     ▢ Did not occur
     ▢ Occurred, but it did not affect me at all
     ▢ Occurred, and it affected me a little
     ▢ Occurred, and it affected me a lot
14. During the childbirth experience, I was made to feel vulnerable, guilty, insecure, or that I had not lived up to what was expected of me (that I had not “cooperated”).
     ▢ Did not occur
     ▢ Occurred, but it did not affect me at all
     ▢ Occurred, and it affected me a little
     ▢ Occurred, and it affected me a lot
15. I was allowed to adopt the position I requested during dilation and delivery, since there were no medical reasons preventing it.
     ▢ Yes, I was allowed
     ▢ No, but it did not affect me at all
     ▢ No, and it affected me a little
     ▢ No, and it affected me a lot
16. Anesthesia was used, either requested or not, for example, to suture a tear or episiotomy (vaginal cut) or to manually remove the placenta.
     ▢ Yes, it was used
     ▢ No, but it did not affect me at all
     ▢ No, and it affected me a little
     ▢ No, and it affected me a lot
17. Vaginal examinations were performed without taking measures to reduce discomfort (such as using lubricant, performing the technique gradually, or helping me relax).
     ▢ Yes, such measures were used
     ▢ No, but it did not affect me at all
     ▢ No, and it affected me a little
     ▢ No, and it affected me a lot
18. Some of these procedures were performed without my approval (enema, shaving, vaginal examinations, episiotomy, abdominal pressure).
     ▢ Yes, with my approval
     ▢ Performed without my approval, but it did not affect me at all
     ▢ Performed without my approval, and it affected me a little
     ▢ Performed without my approval, and it affected me a lot
19. I suffered some type of physical aggression during childbirth, for example, I was slapped on the face or thighs to scold or reprimand me for my behavior.
     ▢ Did not occur
     ▢ Occurred, but it did not affect me at all
     ▢ Occurred, and it affected me a little
     ▢ Occurred, and it affected me a lot

20.a I was allowed immediate skin-to-skin contact after birth without medical reasons against it and/or with no explanations given to the contrary (only for women whose babies were born alive).
 ▢ Yes, I was allowed
 ▢ No, but it did not affect me at all
 ▢ No, and it affected me a little
 ▢ No, and it affected me a lot

20.b I was offered the possibility of seeing my baby or preparing a memory box (only for women who experienced a fetal loss).
 ▢ Yes, I was offered
 ▢ No, but it did not affect me at all
 ▢ No, and it affected me a little
 ▢ No, and it affected me a lot

## ****SECTION 3.****

**Edinburgh Postnatal Depression Scale (EPDS)**

**Instructions:**
We would now like to know how you have felt after childbirth.
Please mark the option that best describes how you have felt **during the past 7 days**, not just today.
For example: “I have felt happy most of the time during the past week.”
Please answer the other questions in the same way.
(An asterisk indicates that the question is mandatory.)

**I have been able to laugh and see the funny side of things***
▢ As much as I always could
▢ Not quite so much now
▢ Definitely not so much now
▢ Not at all

**I have looked forward with enjoyment to things***
▢ As much as ever
▢ Rather less than I used to
▢ Definitely less than I used to
▢ Hardly at all

**I have blamed myself unnecessarily when things went wrong***
▢ Yes, most of the time
▢ Yes, some of the time
▢ Not very often
▢ No, never

**I have been anxious or worried for no good reason***
▢ No, not at all
▢ Hardly ever
▢ Yes, sometimes
▢ Yes, very often

**I have felt scared or panicky for no very good reason***
▢ Yes, quite a lot
▢ Yes, sometimes
▢ No, not much
▢ No, not at all

**Things have been getting on top of me***
▢ Yes, most of the time I haven’t been able to cope
▢ Yes, sometimes I haven’t been coping as well as usual
▢ No, most of the time I have coped quite well
▢ No, I have been coping as well as ever

**I have been so unhappy that I have had difficulty sleeping***
▢ Yes, most of the time
▢ Yes, sometimes
▢ Not very often
▢ No, not at all

**I have felt sad or miserable***
▢ Yes, most of the time
▢ Yes, quite often
▢ Not very often
▢ No, not at all

**I have been so unhappy that I have been crying***
▢ Yes, most of the time
▢ Yes, quite often
▢ Only occasionally
▢ No, never

**The thought of harming myself has occurred to me***
▢ Yes, quite often
▢ Sometimes
▢ Hardly ever
▢ Never

## ****SECTION 4.****

**Perinatal Post-Traumatic Stress Disorder Questionnaire (PPQ)**

**Instructions:**
Below, we ask you to assess how often you have experienced the following situations.
Please mark the option that best reflects your experience.

**Response scale:**
Nothing – Once or twice – Often but for less than one month – Often and for more than one month

| **Situation** | **Nothing** | **Once or twice** | **Often but for less than one month** | **Often and for more than one month** |
| --- | --- | --- | --- | --- |
| I have had dreams or nightmares related to the birth of my baby. | ☐ | ☐ | ☐ | ☐ |
| Thinking about my childbirth or my baby’s hospital stay brings back bad memories. | ☐ | ☐ | ☐ | ☐ |
| I have had the sudden feeling that my childbirth was happening again (flashback). | ☐ | ☐ | ☐ | ☐ |
| I try or have tried to avoid thinking about things related to the birth of my baby. | ☐ | ☐ | ☐ | ☐ |
| I avoid doing things that remind me of the birth or the hospital stay. | ☐ | ☐ | ☐ | ☐ |
| I have gaps in my memory or difficulty remembering moments from my hospital stay. | ☐ | ☐ | ☐ | ☐ |
| I feel less interested in activities that I used to enjoy. | ☐ | ☐ | ☐ | ☐ |
| I feel lonely or detached from others. | ☐ | ☐ | ☐ | ☐ |
| I have found it difficult to feel affection or love for those around me. | ☐ | ☐ | ☐ | ☐ |
| I am having trouble sleeping. | ☐ | ☐ | ☐ | ☐ |
| I have felt more angry with others than before. | ☐ | ☐ | ☐ | ☐ |
| I have had trouble concentrating since the birth. | ☐ | ☐ | ☐ | ☐ |
| I feel more irritable (for example, noise bothers me more than usual). | ☐ | ☐ | ☐ | ☐ |
| I feel more guilty about the circumstances surrounding the birth of my baby than I think is normal. | ☐ | ☐ | ☐ | ☐ |

## ****SECTION 5.****

**Quality of Lifen Questionnaire**

**The following questions refer to what you think about your health. Please answer each question by marking one box. If you are not sure how to answer a question, please choose the response that seems most accurate to you.**

### ****In general, would you say your health is:*****

Select one oval.
▢ Excellent
▢ Very good
▢ Good
▢ Fair
▢ Poor

The next questions refer to activities or things you might do on a normal day. **Does your current health limit you in these activities? If so, how much?**

### ****Moderate activities such as moving a table, vacuuming, bowling, or walking for more than 1 hour:*****

Select one oval.
▢ Yes, it limits me a lot
▢ Yes, it limits me a little
▢ No, it does not limit me at all

### ****Climbing several flights of stairs:*****

Select one oval.
▢ Yes, it limits me a lot
▢ Yes, it limits me a little
▢ No, it does not limit me at all

During the past 4 weeks, **have you had any of the following problems in your work or daily activities because of your physical health?**

### ****Did you accomplish less than you would have liked?*****

Select one oval.
▢ Yes
▢ No

### ****Did you have to stop doing some tasks at work or in your daily activities?*****

Select one oval.
▢ Yes
▢ No

During the past 4 weeks, **have you had any of the following problems in your work or daily activities because of emotional problems (such as feeling sad, depressed, or nervous)?**

### ****Did you accomplish less than you would have liked because of an emotional problem?*****

Select one oval.
▢ Yes
▢ No

### ****Did you not perform your work or daily activities as carefully as usual because of an emotional problem?*****

Select one oval.
▢ Yes
▢ No

### ****During the past 4 weeks, to what extent has pain interfered with your normal work (including work outside the home and housework)?*****

Select one oval.
▢ Not at all
▢ A little
▢ Moderately
▢ Quite a bit
▢ Very much

The following questions refer to how you have felt and how things have been for you during the past 4 weeks.
For each question, choose the answer that comes closest to how you have felt.

### ****During the past 4 weeks, how often…****

### ****… did you feel calm and peaceful?*****

Select one oval.
▢ Always
▢ Almost always
▢ Often
▢ Sometimes
▢ Rarely
▢ Never

### ****… did you have a lot of energy?*****

Select one oval.
▢ Always
▢ Almost always
▢ Often
▢ Sometimes
▢ Rarely
▢ Never

### ****… did you feel downhearted and sad?*****

Select one oval.
▢ Always
▢ Almost always
▢ Often
▢ Sometimes
▢ Rarely
▢ Never

### ****During the past 4 weeks, how often have physical health or emotional problems interfered with your social activities (such as visiting friends or relatives)?*****

Select one oval.
▢ Always
▢ Almost always
▢ Often
▢ Sometimes
▢ Rarely
▢ Never

### ****Do you consider that your current health condition is related to your childbirth experience?*****

Select one oval.
▢ No
▢ Yes, partially
▢ It is mainly due to my pregnancy, childbirth, and postpartum experience

## ****SECTION 6.****

# ****Detection of Gender Violence****

The following questions relate to tools for the early detection of gender-based violence

### ****In general, how would you describe your relationship with your partner?*****

Select one oval.
▢ A lot of tension
▢ Some tension
▢ No tension

### ****You and your partner resolve your disagreements with…*****

Select one oval.
▢ A lot of difficulty
▢ Some difficulty
▢ No difficulty

## ****SECTION 7.****

# ****Sexual Function (FSFI-type section)****

**The following questions concern your sexual feelings and responses during the past 4 weeks. Please answer as honestly and clearly as possible. We will define some terms:**

- **Sexual activity:** includes caressing, sexual play, masturbation, and vaginal intercourse.
- **Vaginal intercourse:** insertion of the penis into the vagina.
- **Sexual stimulation:** includes sexual play with a partner, self-stimulation (masturbation), or sexual fantasies.
- **Sexual desire or interest:** includes the wish to have a sexual experience, feeling receptive to a partner’s sexual initiation, and having sexual thoughts or fantasies.
- **Sexual arousal:** includes physical and mental aspects of sexual excitement. It may include warmth or throbbing in the genitals, lubrication (wetness), or muscle contractions.

### ****During the past 4 weeks, how often did you experience sexual desire or interest?*****

Select one oval.
▢ Almost always or always
▢ Most of the time (more than half the time)
▢ Sometimes (about half the time)
▢ A few times (less than half the time)
▢ Almost never or never

### ****During the past 4 weeks, how would you rate your level of sexual desire or interest?*****

Select one oval.
▢ Very high
▢ High
▢ Moderate
▢ Low
▢ Very low or none

### ****During the past 4 weeks, how often did you feel sexually aroused during sexual activity or vaginal intercourse?*****

Select one oval.
▢ No sexual activity
▢ Almost always or always
▢ Most of the time (more than half the time)
▢ Sometimes (about half the time)
▢ A few times (less than half the time)
▢ Almost never or never

### ****During the past 4 weeks, how would you rate your level of sexual arousal during sexual activity or vaginal intercourse?*****

Select one oval.
▢ No sexual activity
▢ Very high
▢ High
▢ Moderate
▢ Low
▢ Very low or none

### ****During the past 4 weeks, how confident were you in becoming sexually aroused during sexual activity or vaginal intercourse?*****

Select one oval.
▢ No sexual activity
▢ Very high confidence
▢ High confidence
▢ Moderate confidence
▢ Low confidence
▢ Very low confidence or none

### ****During the past 4 weeks, how often were you satisfied with your sexual arousal during sexual activity or vaginal intercourse?*****

Select one oval.
▢ No sexual activity
▢ Almost always or always
▢ Most of the time (more than half the time)
▢ Sometimes (about half the time)
▢ A few times (less than half the time)
▢ Almost never or never

### ****During the past 4 weeks, how often did you achieve vaginal lubrication (vaginal wetness) during sexual activity or vaginal intercourse?*****

Select one oval.
▢ No sexual activity
▢ Almost always or always
▢ Most of the time (more than half the time)
▢ Sometimes (about half the time)
▢ A few times (less than half the time)
▢ Almost never or never

### ****During the past 4 weeks, how difficult was it for you to become lubricated (to have vaginal wetness) during sexual activity or vaginal intercourse?*****

Select one oval.
▢ No sexual activity
▢ Extremely difficult or impossible
▢ Very difficult
▢ Difficult
▢ Slightly difficult
▢ Not difficult

### ****During the past 4 weeks, how often did you maintain vaginal lubrication (vaginal wetness) until completion of sexual activity or vaginal intercourse?*****

Select one oval.
▢ No sexual activity
▢ Almost always or always
▢ Most of the time (more than half the time)
▢ Sometimes (about half the time)
▢ A few times (less than half the time)
▢ Almost never or never

### ****During the past 4 weeks, how difficult was it for you to maintain vaginal lubrication (vaginal wetness) until completion of sexual activity or vaginal intercourse?*****

Select one oval.
▢ No sexual activity
▢ Extremely difficult or impossible
▢ Very difficult
▢ Difficult
▢ Slightly difficult
▢ Not difficult

### ****During the past 4 weeks, when you experienced sexual stimulation or vaginal intercourse, how often did you reach orgasm (climax)?*****

Select one oval.
▢ No sexual activity
▢ Almost always or always
▢ Most of the time (more than half the time)
▢ Sometimes (about half the time)
▢ A few times (less than half the time)
▢ Almost never or never

### ****During the past 4 weeks, when you experienced sexual stimulation or vaginal intercourse, how difficult was it for you to reach orgasm (climax)?*****

Select one oval.
▢ No sexual activity
▢ Extremely difficult or impossible
▢ Very difficult
▢ Difficult
▢ Slightly difficult
▢ Not difficult

### ****During the past 4 weeks, how satisfied were you with your ability to reach orgasm (climax) during sexual activity or vaginal intercourse?*****

Select one oval.
▢ No sexual activity
▢ Very satisfied
▢ Moderately satisfied
▢ Neither satisfied nor dissatisfied
▢ Moderately dissatisfied
▢ Very dissatisfied

### ****During the past 4 weeks, how satisfied were you with the emotional closeness between you and your partner during sexual activity?*****

Select one oval.
▢ No sexual activity
▢ Very satisfied
▢ Moderately satisfied
▢ Neither satisfied nor dissatisfied
▢ Moderately dissatisfied
▢ Very dissatisfied

### ****During the past 4 weeks, how satisfied were you with your sexual relationship with your partner?*****

Select one oval.
▢ Very satisfied
▢ Moderately satisfied
▢ Neither satisfied nor dissatisfied
▢ Moderately dissatisfied
▢ Very dissatisfied

### ****During the past 4 weeks, how satisfied were you with your sexual activity overall?*****

Select one oval.
▢ Very satisfied
▢ Moderately satisfied
▢ Neither satisfied nor dissatisfied
▢ Moderately dissatisfied
▢ Very dissatisfied

### ****During the past 4 weeks, how often did you experience discomfort or pain during vaginal intercourse?*****

Select one oval.
▢ No vaginal intercourse
▢ Almost always or always
▢ Most of the time (more than half the time)
▢ Sometimes (about half the time)
▢ A few times (less than half the time)
▢ Almost never or never

### ****During the past 4 weeks, how often did you experience discomfort or pain after vaginal intercourse?*****

Select one oval.
▢ No vaginal intercourse
▢ Almost always or always
▢ Most of the time (more than half the time)
▢ Sometimes (about half the time)
▢ A few times (less than half the time)
▢ Almost never or never

### ****During the past 4 weeks, how would you rate your level of pain or discomfort during or after vaginal intercourse?*****

Select one oval.
▢ No vaginal intercourse
▢ Very high
▢ High
▢ Moderate
▢ Low
▢ Very low or none

### ****After childbirth, how long did it take for you to resume sexual intercourse?*****

Select one oval.
▢ Less than one month
▢ Between one and two months
▢ Between two and three months
▢ Between 3 and 6 months
▢ Between 6 months and one year
▢ I have not resumed sexual intercourse yet
